# A Method to Measure the Diffusion Coefficient in Liquids

**DOI:** 10.1007/s11242-021-01704-0

**Published:** 2021-10-20

**Authors:** Mayumi Hamada, Pietro de Anna

**Affiliations:** Institute of Earth Sciences, University of Lausanne, 1015 Lausanne, Switzerland

**Keywords:** Diffusion, Confinement, Mixing

## Abstract

Molecular diffusion in liquids is a key process in numerous systems: it is often the reaction rate limiting factor in biological or chemical reaction. Molecular diffusion has been recognized as the ultimate mechanism by which substances concentration get homogenized and, thus, their mixing and dilution occur. Here, we propose a novel method to directly measure the diffusion coefficient *D* of solutes or suspensions in liquids. Differently from current methods, as Dynamic Light Scattering or Fluorescent Correlation Spectroscopy, our method does not rely on previous knowledge on the fluid or tracer properties, but it is based on directly measuring the concentration spatial profile of a considered tracer with optical techniques within a diffusion chamber. We test this novel method on a sample of mono-dispersed suspension of spherical colloids for which an estimate for *D* can be made based on Einstein–Stokes relation. We, then, use this technique to measure the diffusion coefficient of a non-spherical tracer. We further quantify mixing of the considered tracers in the confined domain of the diffusion chamber: we show that, since diffusion-limited mixing (quantified in terms of the dilution index) in a confined space happens faster than un-confined domain, the finite size of the diffusion chamber must be taken into account to properly estimate *D* and the tracer mixing degree.

## Introduction

Molecular diffusion in liquids is a key process in numerous natural and engineering systems (Graham 1849; Dentz et al. 2011). It is often the reaction rate limiting factor in biological or chemical reactions (de Anna et al. 2014a, b). Generally, it is the ultimate mechanism by which substances concentration get homogenized and, thus, their mixing and dilution occur (Ottino 1989; Villermaux 2019; Le Borgne et al. 2015). Molecular diffusion of a given dissolved or suspended compound originates from the individual molecules (or particles) motion that is associated to their thermal agitation: a famous example is the early observation of pollen grains movement in water by Pas (1971): the macroscopic consequence of this microscopic phenomenon is that the mass of that compound spreads in space as time passes.

The description of the macroscopic spreading of a compound *c* under diluted conditions is given by Fick’s first law: it states, in analogy with Fourier’s law of thermal conductivity, that the diffusive mass flux *J*(*x*) at a location *x* is proportional to the concentration gradient

1 $$\begin{aligned} J(x) = - D \frac{\partial c(x)}{\partial x} \end{aligned}$$

where the constant of proportionality *D* is the so-called diffusion coefficient. The negative sign implies that mass moves from locations with higher concentration towards areas of lower concentrations. Since their gradient changes with time as the substance diffuses, mass conservation must be invoked to describe the concentration spatio-temporal dynamics:

2 $$\begin{aligned} \frac{\partial c(x,t)}{\partial t} = - \frac{\partial J(x,t)}{\partial x}. \end{aligned}$$

It states that for a given location *x*, a change in the mass flux is associated with a change of concentration in time. Combining the two Fick’s laws, we obtain the well-known diffusion equation describing the spatio-temporal distribution of a diffusing substance:

3 $$\begin{aligned} \frac{\partial c(x,t)}{\partial t} = - D \, \frac{\partial ^2 c(x,t)}{\partial x^2}. \end{aligned}$$

The knowledge of *D* is crucial to describe the fate of a diffusing substance and all the diffusion-related phenomena, like mixing or reactions. For spherical objects, the value of the diffusion coefficient can be theoretically derived from the well-known Stokes–Einstein relation (Reif 1985) which compares the velocity associated to the kinetic energy of particles to the viscous drag experienced, by the particles themselves, while moving within a fluid of viscosity $\mu$. It reads:

4 $$\begin{aligned} D = \frac{k\,T}{6\,\pi \,\mu \,r}, \end{aligned}$$

where *k* is the Boltzmann constant [J/K], *T* is the absolute temperature [K], $\mu$ is the dynamic viscosity [Pa$\,s$] and *r* is the particles radius [m]. For objects of approximately spherical shape (e.g. many types of molecules, colloids or bacteria) for which the radius is known, several methods have been developed in the past decades to measure the value of *D* based either on the microscopic (individual motion) or macroscopic (concentration distribution) properties of the process.

Dynamic Light Scattering (DLS) measures intensity fluctuation of light scattered by particles and relates it to the particle velocity. It is a technique typically used to determine the size distribution of particles in suspension. It assumes quasi-elastic scattering of light by a homogeneous set of spherical objects of similar diameter. Measuring the size of diffusing particles, based on Eq. (4), the diffusion coefficient can be calculated (Stetefeld et al. 2016).

Fluorescent Correlation Spectroscopy (Yu et al. 2021) is another widely used method to estimate *D* by measuring the temporal autocorrelation of the detected fluorescence signal emitted by a volume which is tiny and controlled (typically via confocal microscopy) of liquid containing a well diluted compound. Due to the extremely short time range over which the autocorrelation is measured (from microsecond to second) and the tiny signal emitted, the light must be detected by a fast acquisition device as a photomultiplier, an avalanche photodiode or a superconducting nanowire single-photon detector. The measured decay of the signal autocorrelation reveals the time needed by a molecule to diffuse through the observation volume of linear size *a*: this time scale is expected to be $\tau _D = a^2/4D$. If *a* is known and $\tau _D$ is measured, *D* can be estimated.

Other methods to measure the diffusion coefficient *D* in liquids are based on macroscopic mass transfer. For instance, the one based on Taylor dispersion within a Poiseuille flow, where a pulse of a substance is injected within a tube stream and the concentration measured at the outlet. The obtained profile is then fitted to the solution of dispersion equation where the proportionality constant $D_t$ is Taylor diffusivity. The value of the diffusion coefficient *D* can be then back computed knowing the tube radius *r* and mean flow velocity *u* through $D_t = r^2\,u^2/(48\,D)$ (Alizahed et al. 1980; Ouano 1972).

Another method exploits the diaphragm cell (Northrop and Anson 1928; Gordon 1945; Lozar et al. 1975): two reservoirs of volume *V* are separated by a porous membrane and a solute diffuses from one to the other through the membrane. The concentration is measured in one reservoir at time interval *dt* and thus the rate of change of solute concentration $dc/dt = (c_2 - c_1)/(t_2 - t_1)$ in the reservoir is given by Fick’s law and depends on the membrane width *l* and effective porosity *A*, from which the value of *D* is determined. A calibration with a solute of known diffusion coefficient is required to determine *A*.

All these methods are i) based on indirect measurements or ii) require previous knowledge on both solute and solvent properties or iii) require an expensive and hard to use/calibrate instrumentation. We propose, here, a novel and simple method to measure the diffusion coefficient *D* that, with the proposed set-up, has uncertainty of about $3\%$ and requires no prior knowledge on either the target substance or on its solvent.

## Method

Let us consider a tracer of concentration *c* dissolved, or suspended, in a given liquid. The main idea behind our method is to measure the spatio-temporal evolution of the concentration profile *c*(*x*, *t*) with optical techniques, under initial and boundary conditions for which an analytical solution of the diffusion equation Eq. (3), depending only on *D*, is known. By fitting this analytical solution *c*(*x*, *t*) to the measured concentration profile will provide an estimate of the diffusion coefficient *D*. To validate our experimental set-up, we use a tracer for which the diffusion coefficient can be predicted by the Stokes–Einstein relation (Reif 1985): we choose a mono-dispersed suspension of fluorescent micro-spheres. We will, then, apply the same methodology to a colored tracer whose molecule is non-spherical.

### Fluorescent Spheres as Tracer

We use polystyrene fluorescent micro-spheres (Fluoro-Max, Thermo Fisher B150) of radius $r = 0.075 \, \mu m$ that are provided at $1\%$ solid concentration. From the original suspension, we prepare a concentration $c_0$ 20 times diluted in a milliQ-water and heavy-water ($D_2O$,) mixture of density 1.05 g/ml, matching the one of the micro-spheres to avoid their sedimentation. With the optical system used, the particles are too small to be individually detected. Instead, we observe the overall fluorescent signal emitted by the suspension within the field of view. A calibration procedure showed us that the amount of light detected and recorded by our acquisition system is proportional to the tracer concentration in the range $[0,c_0]$.

The light detected by the camera is recorded into a greyscale image and stored as a matrix *im* of integer values between 0 (black) and $2^{bit} - 1$ (white), where *bit* represents the color depth of the camera. We used a Nikon DS-Qi2 which is equipped with a CMOS full-frame sensor recording at 12-bit. If the tracer is not so concentrated to block part of the incoming and its own emitted light, the value of this matrix *im* is proportional to the tracer concentration as $im = s\,c + im_B$, where *s* is a proportionality constant and $im_B$ represents the background signal detected in absence of tracer, $c = 0$. Thus,

5 $$\begin{aligned} c = \frac{1}{s} \, (im - im_B), \end{aligned}$$

where the value of *s* can be found via a calibration procedure collecting pictures of samples of known concentration. We verified via a calibration that the tracer at the adopted concentration satisfies Eq. (5): however, to avoid propagation of error associated to the estimation of the parameter *s*, we express the concentration *c* relative to its initial value $c_0$:

6 $$\begin{aligned} \frac{c}{c_0} = \frac{im - im_B}{im_0 - im_B}, \end{aligned}$$

where $im_0$ is the matrix representing image collected when only the tracer at concentration $c_0$ is present, so that $c/c_0$ does not depend on the estimation of the parameter *s* or the initial concentration $c_0$.

### Colored Dye as Tracer

The second tracer we use is a solution of methyl blue dye (Sigma-Aldrich) of concentration $c_0 = 0.15$ mg/l. The solution is prepared with a mixture of milliQ water (80$\%$) and glycerol (20$\%$). Once a sample of this solution is irradiated with light (bright field microscopy), only a portion of the signal passes through while portion of it is absorbed. The more concentrated is the tracer, the more light is absorbed and the less of it is transmitted and detected. The light absorbance, the logarithm of the ratio between incoming and transmitted light, is a linear function of the tracer concentration according to the Beer-Lambert law (Bouguer 1729). The exponential dependence of the transmitted light to the concentration can be simplified as linear for low concentrations, so that:

7 $$\begin{aligned} \frac{c}{c_0} = \frac{im_B - im}{im_B - im_0}, \end{aligned}$$

where $im = im(c)$ is the transmitted light intensity through the tracer at concentration *c* (and detected by the camera), $im_0 = im(c = c_0)$ and $im_B = im(c = 0)$.

**Fig. 1:**
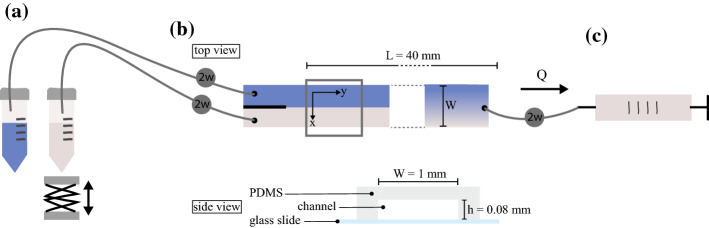
Set-up for diffusion experiments where we optically measure the diffusion profile of a solute tracer in a microfluidic channel. *a*. Reservoirs for tracer and blank solution, one reservoir is placed on a laboratory jack to adjust water level and ensure equal head between the two; *b*. Side view: cross section of a microfluidic chip male of a PMS channel sealed to a microscopy glass slide, the channel dimensions are height *h* = 0.08 mm, width *w* = 1 mm; top view of the channel: length *L* = 40 mm, the grey rectangle indicates the position of image acquisition, in the y direction, fluids have a velocity $u_y$, in the x direction, $u_x = 0$ and molecules are displaced by diffusion only, close to the inlet, the front between the tracer and blank is sharp while further downstream it is more diffused; *c*. Syringe pump in withdraw mode creates a flow *Q* in the channel, the flow direction in the system is indicated by the black arrow. Reservoirs, channel and syringe are connected using Tygon tubes

### Diffusion Chamber

In order to reproduce the conditions for which a tracer is diffusing along one dimension and compare its concentration profile to the solution of Eq. (3), we build a microfluidics device (used as diffusion chamber). In it, we continuously inject, side by side, the considered tracer solution/suspension and its solvent (in the following called blank solution). Thus, we design a channel mold with rectangular cross section and a parallel injection entrance (Fig. 1). In this flow cell, the solutions flow along the channel longitudinal, main, direction only. The mass transfer mechanisms taking place along the transverse direction is molecular diffusion alone. The cell geometry is printed onto transparent glass at high resolution in chrome (JD Photodata, UK). Micro-channels are fabricated using standard techniques of soft lithography and PDMS molding. They are then plasma-bonded to a 1-mm-thick soda-lime glass slide. The resulting channel has width $w = 1$ mm, thickness $h = 0.08$ mm (thus rectangular cross section area $A = 0.08$ mm$^2$) and a length $L = 40$ mm (see Fig. 1).

### Flow System

Each inlet is connected with Tygon tubing (internal diameter of 0.5 mm) to a reservoir (15 ml Falcon tubes). One contains 4 ml of the blank solution, the other contains 4 ml of tracer solution. The outlet is connected to a waste reservoir containing 4 ml of water. Tubing connecting the microfluidic chip to the reservoirs can be open/closed at will by means of 2-ways microfluidic valves (MaxWire from Elveflow), all three reservoirs are pressurized using a pressure controller (OB1 MK3+ from Elveflow) so that the flow is established by a pressure drop between inlet and outlet of $\Delta p = 50$ mbar. Once the flow is interrupted (by closing simultaneously all valves and stopping the pressure drop), the tracer diffuses transversely towards the blank solution. In this configuration, the one-dimensional tracer concentration profile along the channel transverse direction is the solution of Eq. (3).

### Optical System and Image Processing

**Fig. 2:**
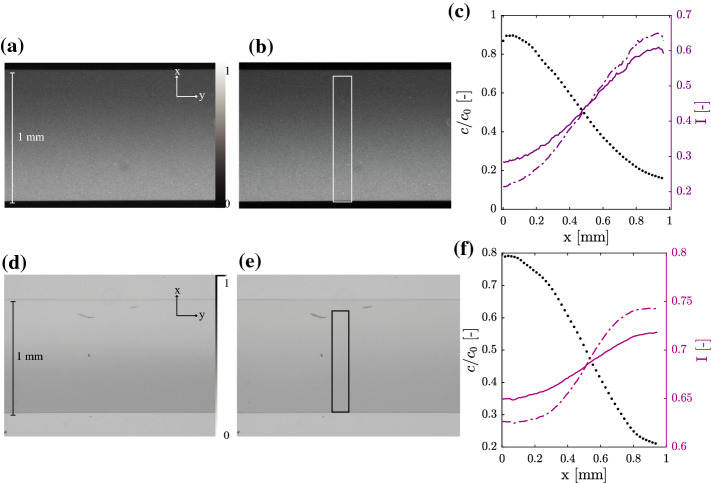
Microscopy images of the channel with 15X magnification. Fluids are flowing along the horizontal, *y*-direction, and tracer diffuses along the transverse *x*-direction. Images $im_0$ corresponding to $c = c_0$ (*a* for fluorescent spheres and *d* for colored dye) and $im_B$ corresponding to $c = 0$ (*b* for fluorescent spheres and *e* for colored dye). The profiles (*c* for fluorescent spheres and *f* for colored dye) are obtained by summing pixel values within the white rectangle along the y-direction. On the right scale, light intensity profile of $im_0$ (dashed line) and *im* (solid line); on the left scale, the associated concentration profile Eq. (6) for spheres and Eq.(7) for colored dye

The microfluidic device is placed under a microscope (an inverted Nikon Eclipse Ti-E2) equipped with a low numerical aperture (NA = 0.3) objective in order to observe in focus the whole depth of the channel. On the one hand, for imaging the fluorescent particles, excitation and emission light are selected using a filter-cube (Nikon, DAPI, excitation bandpass $395 \pm 10$ nm and emission bandpass $475 \pm 11$ nm). On the other hand, for imaging the methyl blue solute, a custom filter selects the irradiating white light (Semrock single-band band pass filter $662 \pm 11$ nm), so that only near-blue light reaches the sample, the one that is the most absorbed. For all cases, greyscale images are captured and stored using a Nikon DS-Qi2 camera. Each image is composed by $4908 \times 3264$ pixels whose physical size in the camera sensor is $7.3 \, \mu$m: thus, considering the objective magnification used (objective 10X plus the internal microscope 1.5X extra magnification, for a total of 15X), an overall size of $2.3 \times 1.6$ mm. The images acquired are matrices of pixels whose value ranges from 0 to $2^{12} - 1$. We crop each image (*im*, $im_0$ and $im_B$) to a desired region of interest (rectangles in Fig. 2a and b) which goes from wall to wall of the microfluidic and spans 300 pixels longitudinally (along the flow direction, *y*) and we compute its profile (Fig. 2c) by averaging values along *y*-direction. Finally, for both tracers (fluorescent spheres and colored dye), the concentration profiles are obtained from the above equations where *im*, $im_0$ and $im_B$ are the profiles of the considered pictures.

### Theoretical Estimate of *D*

The polystyrene particles diffusion coefficient *D* can be theoretically estimated with the Stokes–Einstein relation (Reif 1985), Eq. (4). Working at $T = 293$ K with a suspension of viscosity $\mu = 1.06 \, 10^{-3}$ Pa s and particles of size *r* = 7.5$\,10^{-8}$ m, the diffusion coefficient is estimated as $D_b = 2.7 \, 10^{-6}$ mm$^2$/s.

Methyl blue molecules present a non-spherical structure, closer to a sheet (much thinner than wide), thus, we define an effective radius $\overline{r}$ = $6.5\,10^{-10}$ m (Kipling and Wilson 1960; Hang and Brindley 1970; Taylor 1985). The dynamic viscosity of the water-glycerol mixture is $\mu _m$ = 1.98 Pa s and, thus, we estimate $D_m = 1.7 \, 10^{-4}$ mm$^2$/s.

### Solution of Diffusion Equation

Since the fluid flow is stopped by closing the valves, the only mechanism of mass transfer is molecular diffusion. For the experimental configuration chosen, the only spatial direction along which a nonzero macroscopic tracer gradient and, thus, a mass flux takes place is the transverse one (denoted as *x* in Fig. 1). The tracer spreads between the microfluidics boundaries, i.e the PDMS walls: the concentration profile that we measure is the solution of the one-dimensional diffusion equation (Eq. (3)), with no-flux boundary conditions at $x=0,L$, as derived in (Hamada et al. 2020):

8 $$\begin{aligned} \frac{c(x,t)}{c_0} = \sum _{m = 1}^{\infty }\,B_m\,\cos (\pi \,m\,x/L)\,e^{-\pi ^2\,m^2\,D \, t/L^2} + c_f. \end{aligned}$$

where $c_f = 1 / L$ is the homogeneous concentration reached at times much larger than the characteristic diffusive time scale over the channel width $t > \tau _D = L^2 / D$ and $B_m$ is a coefficient that depends on the initial concentration distribution $f_0(x)$:

9 $$\begin{aligned} B_m = 2\,\int _0^1 f_0(x)\,\cos (\pi \,m\,x)\,dx. \end{aligned}$$

Note that, the initial condition $f_0(x)$ corresponds to the concentration profile collected at any given time $t_0$ ($f_0(x) = c(x,t_0)$) for which it will be imposed that $t_0 = 0$: the initial profile can be chosen at convenience. The characteristic time by which molecular diffusion smooth the concentration gradient, homogenizing the concentration field, over le the length scale *L* is commonly considered to be $\tau _D = L^2/D$. However, for a confined condition, i.e. no-flux at boundaries, the solution of the diffusion equation is Eq. (8) which is the superposition of modes *m* that decay exponentially fast as $e^{-\pi ^2 m^2 D t / L^2}$. For the case of a diffusive front, as considered here, the smallest nonzero mode is $m = 1$ (for a pulse it would be $m=2$): thus, at time larger than $L^2 / (\pi ^2 D) = \tau _D/\pi ^2,$ the whole solution Eq. (8) is dominated by the first mode and it decays exponentially fast with time. Physically, this means that the concentration profile experiences the presence of no-flux boundary conditions that prevent the solute mass to explore more space. In this context, this is relevant since the exponential decay of the solution fixes a temporal scale which is $\tau _D / \pi ^2$. In other words, for larger times, we should expect the concentration profile to be well homogenized within the diffusion chamber. Thus, the measurement must be performed over a shorter time scale: for the beads, $\tau _D / \pi ^2$ is about 10 hours, and for the dye solution, it is about 10 minutes.

We fit, for each time step $t_j$, the analytical solution $c(x_i,t_j)$, Eq. (8), to the measured concentration profile that we label $c_M(x_i,t_j)$ by varying the only parameter *D* until it is reached the minimum of the mean-squared error

10 $$\begin{aligned} \delta c(t_j) = \text {MSE} = \frac{1}{N}\,\sum _{i = 1}^{iN} \left( c(x_i,t_j) - c_M(x_i,t_j) \right) ^2, \end{aligned}$$

where *N* is the number of points over which the concentration profile is detected (number of pixels along the transverse direction within the region of interest). To rigorously assess the uncertainty on *D*, we should estimate how the uncertainty $\delta c$ would propagate on the parameter *D*, by inverting the *c*(*x*, *t*, *D*) into *D*(*x*, *t*, *c*) and computing $\partial D / \partial c$, as $\delta D = \delta c \, |\partial D / \partial c|$. Unfortunately, for two reasons, this is not possible. First, the analytical expression Eq. (8) cannot be inverted due to the sum over the modes *m*, thus the derivative $\partial D / \partial c$ cannot be computed analytically. Second, to estimate the derivative of the inverse of a function as the reciprocal of the function derivative, it is necessary that the function derivative is nonzero. Therefore, the derivative $\partial D / \partial c$ cannot be estimated as the reciprocal of the derivative $(\partial c / \partial D)^{-1}$ since for $m>1$ the derivative $\partial c / \partial D = 0$ at $x = L/m$ within the domain boundaries $0<x<L$.

Therefore, we estimate the measurement uncertainty on the value of *D* as the ratio between the mean $\overline{D}$ and the standard deviation $\sigma$, defined as:

11 $$\begin{aligned} \overline{D} = \frac{1}{n}\sum _{j=0}^{j=n}D_j; \quad \sigma = \sqrt{\frac{1}{n}\,\sum _{j=1}^{j=n}\left( D_j - \overline{D}\right) ^2}, \end{aligned}$$

where *n* is the number of time steps (or samples collected), $D_j$ is the fitted value of *D* at time $t = t_j$.

### Dilution Index

Once the value of diffusion coefficient *D* has been correctly estimated, one can predict the concentration profile *c*(*x*, *t*) at any time and for any initial condition $f_0$ using Eq. 8. The degree of mixing reached by the diffusive system can be described in terms of system entropy or dilution index (Kitanidis 1994):

12 $$\begin{aligned} E(t) = \text {exp}\left( \int c(x,t)\,\,\text {log}(c(x,t))\,dx \right) . \end{aligned}$$

The dilution index *E* increases as the system homogenizes. According to (Hamada et al. 2020), for the initial condition considered in an un-confined system *E* should increase indefinitely as

13 $$\begin{aligned} E(t) = \exp (\sqrt{2 t} I), \qquad I = -\int _{-\infty }^\infty \frac{1}{2} [1 + \text {erf} (y)] \text {ln} \big (\frac{1}{2} [1 + \text {erf} (y)] \Big ) dy. \end{aligned}$$

However, in a confined scenario, as in a single pore or in our microfluidics system, as soon as the concentration profile experiences the presence of the impermeable boundaries (i.e. for times larger than $\tau _D/\pi ^2$, as discussed in (Hamada et al. 2020), *E* should deviate from the scaling $\text {exp}(\sqrt{t})$ to plateau at $\sqrt{2}$ (or $\text {ln}(E) \sim 0.346$) once the concentration profile get homogenized and the system is well mixed.

## Results

### Polystyrene Fluorescent Particles

**Fig. 3:**
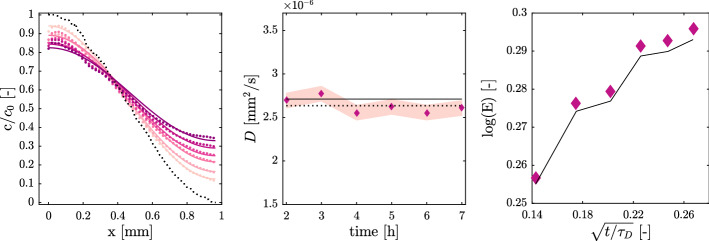
**a** Laboratory measurement of the diffusive concentration profile of polystyrene particles suspension (dots) for six time steps (t = $7.5\,10^{-2}$ to $2.6\,10^{-1}\,\tau _D$), fitted solution of Eq. (8) (solid lines) with initial profile $f_0(x)$ (black dotted line); **b** Value of fitted diffusion coefficient for each time step (diamonds), mean value $\overline{D}$ (dotted line), theoretical prediction by the Einstein–Stokes relation, Eq. (4) (solid black line), standard deviation $\sigma$ (pink shade area); **c** Dilution index value *E*

We record images of a diffusive front of polystyrene particles over seven hours (about $\tau _D / \pi ^2$, the confined mixing time predicted by (Hamada et al. 2020)) at a rate of one image per hour. The measured concentration profiles are shown as dots in Fig. 3.a, as time increases, the profiles go from light to dark color. The fit of these profiles is superposed as solid lines while the initial condition $f_0(x)$ is shown as black dots. For this data set, the MSE between fitted and measured profiles is on average, over all times, $2.3\,10^{-4}$, corresponding to an average deviation of the analytical model from the measured data of $2\sqrt{2.3 \, 10^{-4}} \sim 0.03$, about $3\%$.

In Fig. 3b (diamonds) are shown the fitted values of *D* for each profile and the average value is $\overline{D} = 2.6\,10^{-6}$mm$^2$/s (Fig. 3b black dotted line). The standard deviation among fitted values, given by Eq. (11), is $\sigma$ = $8.52\,10^{-8}$mm$^2$/s which indicates a deviation around the mean of $3.3\%$. The average value of the measured molecular diffusion coefficient is consistent, within $3\%$ with the theoretical estimation by the Stokes–Einstein relation, showing that the novel method proposed is accurate.

We compute the temporal evolution of the Dilution Index *E*, quantifying the overall mixing degree, as defined in Eq. (12). In Fig. 3c is shown the temporal evolution of $\text {log} [E(t)]$ versus rescaled time $t/\tau _D$ for the measured (diamonds) and fitted (solid line) profile. Note that, the system entropy increases as the particles diffuses, and it will eventually reaches a plateau when the system is completely homogeneous, since no more macroscopic gradients are present and, thus, no more mixing can happen (Hamada et al. 2020). The asymptotic value towards which *E* is approaching results to be $\sqrt{2}$ (or $\text {ln}(E) \sim 0.346$), as predicted by replacing a stationary, flat, concentration profile in Eq. (12). This means that diffusion efficiently mixed the tracer within the confined space of the microfluidics device.

### Methylene Blue Dye

**Fig. 4:**
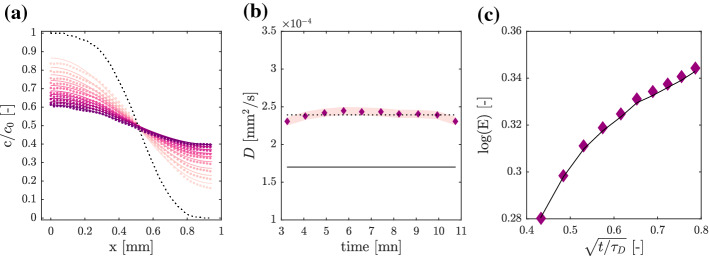
**a**. Laboratory measurement of the diffusive concentration profile of methylene blue (dots) for eleven time steps (t = 0.14 to 0.58$\,\tau _D$), fitted solution of Eq. (8) (solid lines) with initial profile $f_0(x)$ (black dotted line), **b**. Value of fitted diffusion coefficient for each time step (diamonds), mean value $\overline{D}$ (dotted line), standard deviation $\sigma$ (pink shade area), estimation of *D* based on effective radius $\overline{r}$ using the Einstein–Stokes relation Eq.4; **c**. Dilution index value *E*

Images of a methyl blue diffusive front are recorded over 11 minutes (about $\tau _D / \pi ^2$, the confined mixing time predicted by (Hamada et al. 2020)) at a rate of one image every 50 s, the resulting concentration profiles are given in Fig. 4a, as time increases the profiles are shown from light to dark color. We use the first profile (black dots in Fig. 3a) as initial condition $f_0(x)$ for the fit of Eq. (8). The MSE results to be $8.7\,10^{-5}$, corresponding to an average deviation of the analytical model from the measured data of $2\sqrt{8.7 \, 10^{-5}} \sim 0.02$, about $2\%$ and we obtain ten values of fitted diffusion coefficient, one per profile, as shown in Fig. 4b (diamonds).

The average value (dotted line) is $\overline{D} = 2.4\,10^{-4}$mm$^2$/s and the standard deviation among these fitted values, as defined in Eq. (11), is $\sigma$ = $4.89\,10^{-6}$mm$^2$/s which indicates a deviation around the mean of 2 %. The measured value of the diffusion coefficient is 70% higher than the prediction of the Stokes–Einstein relation using the average radius $\overline{r}$. On the one hand, we argue that one of the benefits of the proposed method to measure *D* can be used to provide an estimate of the effective molecule radius *r*, using the Stokes–Einstein relation, eq. (4), which is a robust physical model to estimate *D* for spherical objects suspended in liquid bulk. On the other hand, we have to realize that the effective radius of a non-spherical object could fail in representing it over several applications and, sometimes, it is necessary to avoid that approximation.

We compute the temporal evolution of the Dilution Index *E* for this tracer, as defined in Eq. (12). In Fig. 4c is shown the temporal evolution of $\text {log} [E(t)]$ versus rescaled time $t/\tau _D$ for the measured (diamonds) and fitted (solid line) profile. The result is the same as for the fluorescent micro-spheres: the system mixing degree, its entropy, increases as the tracer diffuses, and it eventually reach a plateau when the microfluidics channel is completely well mixed. The asymptotic value towards which *E* is approaching results to be $\sqrt{2}$ (or $\text {ln}(E) \sim 0.346$), as predicted: diffusion efficiently mixed the tracer within our diffusion chamber.

We verified that the method of fitting the analytical solution of the 1d diffusion equation to the measured concentration profile is robust: we repeated the measurement, for both the particles suspension and the dye solution, using a different camera, withdrawing flow with a syringe pump from two separated reservoirs or infusing them, instead of using a pressure controller, using stainless steal valves (from Swagelock) instead of microfluidics valves, obtaining the same results (same value for *D* within $3\%$ uncertainty).

On the one hand, the main limitation of the proposed methodology is that the substance of interest, the one for which an estimate of *D* is desired, must be detectable and distinguishable from the fluid where it is dissolved/suspended, with optical methods. On the other hand, scientific cameras used to detect the tracer signal are increasingly affordable and relatively easy to use: this makes the proposed method suitable for several applications and, likely, to be developed also for measurements on the field.

As a final remark, we anticipate that it is better to collect pictures under experimental conditions so that the front is not too sharp and not too flat, to avoid large fit uncertainty. If a picture is collected when the solute/suspension diffused a lot across the channel, the tracer profile dependence on space is weak (flat profile). Under these conditions, at a given time $t^*$, a variation in space *x* produces a small variation in $c(x,t^*)$: a variation in the guess value *D*, during the fit procedure, also produces small variation in $c(x,t^*)$ and, thus, a large uncertainty. Note that, also images collected next to the inlet, or with a strong flow rate, result flat for a significant portion (next to the boundaries). In other words, the diffusive front, the portion of space where the profile is not flat, should be as large as possible.

## Conclusions

The novel method presented here allows to measure the diffusion coefficient *D* of a tracer (dissolved or suspended) through a direct visualization of the concentration profile and its dynamics. The profiles measured at different times are fitted with the analytical solution of diffusion equation, with the single fitting parameter *D*. We tested the method measuring the diffusion coefficient of a mono-dispersed suspension of spherical particles for which the Stokes–Einstein relation provides a theoretical estimate. Our results show that the method is accurate: for our test tracer, the discrepancy between measured and theoretical value of the diffusion coefficient is smaller than the method uncertainty of $3\%$.

We show, as reasonably expected, that for non-spherical particles using an effective radius to theoretically estimate the diffusion coefficient can lead to a substantial error: in the case of methyl blue, the value of *D* would be underestimated by $70\%$.

Measuring the concentration profile as it spreads as time passes, we could also estimate the mixing degree of the system: we show experimentally that, as predicted theoretically by (Hamada et al. 2020), under confinement (as in porous systems), diffusion enhances mixing. In absence of confinement (for large domain or for a very short time scale), *E* would keep increasing slowly diluting the tracer: instead, as predicted by (Hamada et al , 2020), in a confined scenario, molecular diffusion is more efficient in mixing. This is quantified by the mixing time scale, the one needed to stop the growth of the dilution index, that is reduced by a factor $1 / \pi ^2 \sim 1/10$ with respect to the characteristic diffusion time $\tau _D = L^2 / D$ over the confinement length scale *L*.
